# Electrogenerated chemiluminescence detection of single entities

**DOI:** 10.1039/d0sc07085h

**Published:** 2021-03-18

**Authors:** Wei Zhao, Hong-Yuan Chen, Jing-Juan Xu

**Affiliations:** State Key Laboratory of Analytical Chemistry for Life Science and Collaborative Innovation Center of Chemistry for Life Sciences, School of Chemistry and Chemical Engineering, Nanjing University Nanjing 210023 China xujj@nju.edu.cn +86-25-89687294 +86-25-89687294

## Abstract

Electrogenerated chemiluminescence, also known as electrochemiluminescence (ECL), is an electrochemically induced production of light by excited luminophores generated during redox reactions. It can be used to sense the charge transfer and related processes at electrodes *via* a simple visual readout; hence, ECL is an outstanding tool in analytical sensing. The traditional ECL approach measures averaged electrochemical quantities of a large ensemble of individual entities, including molecules, microstructures and ions. However, as a real system is usually heterogeneous, the study of single entities holds great potential in elucidating new truths of nature which are averaged out in ensemble assays or hidden in complex systems. We would like to review the development of ECL intensity and imaging based single entity detection and place emphasis on the assays of small entities including single molecules, micro/nanoparticles and cells. The current challenges for and perspectives on ECL detection of single entities are also discussed.

## Abstract 2

We summarize the history and recent development that has been made in the ECL detection of single entities.

## Graphical abstract

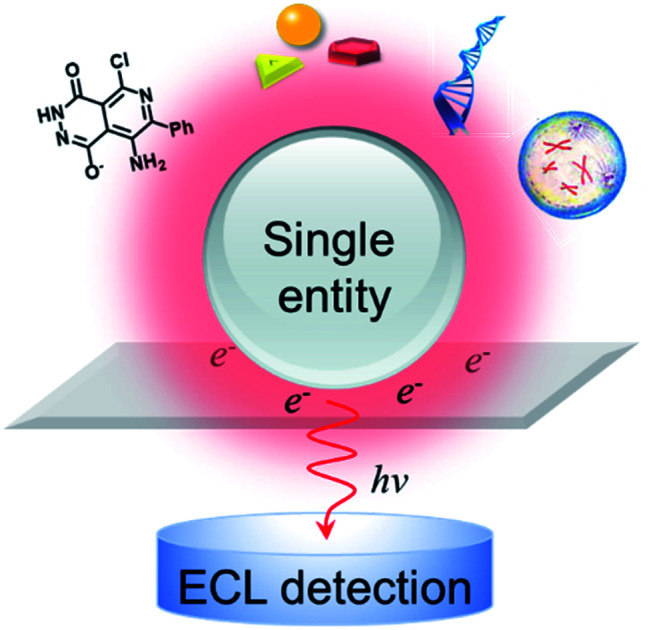

## 1. Introduction

In the past decades, single-entity analysis (SEA) became a prevalent area in analytical chemistry.^1–5^ It refers to the study of individual “entities and processes” which could be a nanoparticle, a molecule, a cell, or a reaction. SEA seeks to separate individual responses from the bulk. It could help us understand the underlying mechanisms of an ensemble object. Since SEA covers many topics and aims at answering questions from different disciplines, it shows promising potential in many fields such as catalysis,^6,7^ DNA sequencing,^8^ and cellular biology.^9,10^ Electrochemistry is the one of the most important methods for SEA because it has the inherent sensitivity requirement for measurements.^11–15^ Nanoscale electrochemical techniques using micro/nano pipettes and electrodes provide fast and low current measurements for the detection of a single-entity. These systems are spatially micro- or nano-confined with high sensitivity but low throughput. Imaging *via* scanning electrochemical microscopy (SECM) and scanning electrochemical cell microscopy (SECCM) made critical applications to SEA measurements.^6,16,17^ Because of the distinctive character of the single-entity, the signal/noise ratio and instrument bandwidth have constantly been challenged by the SEA approaches. It is of great importance to improve the sensitivity, response time, dynamic range and stability of the detecting methods.

Coupling optical measurements with electrochemical processes is an especially compelling approach for SEA.^18–24^ Transformation of the input electrical signal into optical readout would increase the sensitivity and dynamic range of the method. Electrogenerated chemiluminescence, or simply electrochemiluminescence (ECL), is a luminescence phenomenon induced by redox reactions.^25–27^ It is based on the electrochemically generated species that undergo high-energy electron-transfer reactions to form light-emitting excited states. Combining the electrochemical triggering and optical readout, ECL exhibits several unique advantages. Without excitation light, ECL does not suffer from the background interferences, such as autofluorescence and scattered light. In addition, the electrochemical excitation makes the intermediates confined to the vicinity of the electrode surface, which provides both temporal and spatial controllability. Combining these merits, ECL became a powerful tool with remarkable characteristics including near-zero background, fast response speed, and excellent sensitivity.^28,29^ It provides a flexible sensing platform for the applications in many fields, including electrocatalysis,^30^ organic light-emitting diodes,^31^ biosensors,^32,33^ and clinical analysis.^34–36^

The ECL analysis of single entities could be classified into two categories, the intensity and imaging based ECL. In the 1990s, Wightman and co-workers reported ECL from single 9,10-diphenylanthracene molecules in solution.^37^ It is the first account of single-entity ECL based on intensity. The detection was not performed by spatially resolving individual molecules, but temporally recording the asynchronous emission of multiple molecules. In the past 2 decades, the ECL detection of single-entity became more prevalent with the advent of microscopy.^38,39^ As an emerging approach, ECL microscopy (ECLM) exhibits several merits inherited from microscopy, such as good spatiotemporal resolution, high throughput, and low reagent consumption. It promotes the imaging based SEA. Furthermore, it holds advantages including low background and being free of an optical source because of the excitation mode.^40,41^ In addition, without an extrinsic illumination source, the local photothermal effect and other side effects can be eliminated in the system of ECLM.^42^ A growing number of studies regarding the study of single nanoparticles and single cells have been reported based on ECLM.^42,43^

Herein, we review the development of single entities detection using ECL technique as the measuring tool. The mechanistic pathways and setups of ECL are briefly introduced. We focused on the assays of small entities including single molecules, single micro/nanoparticles and single cells. Finally, the challenges and outlooks on ECL detection of single entities are discussed.

## 2. Mechanistic pathways

ECL is the generation of light-emitting excited states formed in the electron transfer processes involving active intermediates obtained during electrochemical reactions. The fundamental ECL generated mechanism is divided into annihilation and coreactant pathways.^44^ In annihilation ECL, both involved reactants (A and B) are directly formed during electrochemical oxidation and reduction. The electrogenerated oxidant and reductant species, A˙^+^ and B˙^−^, then react and produce excited states A* of the luminophore, which relax to the ground state to emit light.^45^ A and B can be the same initial molecule, and then just the redox states are different.

1 A − e → A˙^+^

2 B + e → B˙^−^

3 A˙^+^ + B˙^−^ → A* + B

4 A* → A + *hv*

The early ECL studies were mostly based on the annihilation pathway, which pave the way for the fundamental research of coreactant ECL. The latter has been widely used nowadays for bioanalytical applications.^46,47^ This pathway requires only a single potential step or sweeping the potential in one direction. The coreactant in the system provides energetic radicals that are able to react with the luminophore in order to reach the excited state. The corresponding mechanisms of coreactant ECL are referred to be “oxidative-reduction”^48,49^ and “reductive-oxidation”^50,51^ pathways, respectively. The most common luminophore used in co-reactant “oxidative-reduction” ECL is the Ru(bpy)_3_^2+^/(tri-*n*-propylamine) TPrA pair. In the “oxidative-reduction” ECL, the sequence of reactions generally follows three steps: (1) the oxidation reactions on the electrode surface; (2) bond-breaking or atom-transfer reaction of the coreactant giving a strong reducing radical and (3) the reduction of the oxidized luminophore by the coreactant radical that generates the excited state (eqn (5)–(11)).

5 R − e → R˙^+^

6 C − e → C˙^+^

7 R˙^+^ + C → R + C^+^˙

8 C˙^+^ → C_red_˙

9 C_red_˙ + R → R˙^−^ + P

R˙^+^ + R˙^−^ → R* + Ror

10 C_red_˙ + R˙^+^ → R* + P

11 R* → A + *hv*

R represents the ECL emitter, C represents the co-reactant, C_red_ is the reductive intermediate of the co-reactant, and P represents the product associated with C_red_˙ reactions.

An efficient coreactant for the “reductive-oxidation” path can be strong oxidants (*e.g.* H_2_O_2_ and S_2_O_8_^2−^). In the “reductive-oxidation” pathway, both luminophore and coreactant are first reduced. Then, the reduced form of the coreactant generates a strong oxidizing radical, which further reacts with the reduced luminophore to generate the desired excited state (eqn (12)–(18)).

12 R + e → R˙^−^

13 C + e → C˙^−^

14 R˙^−^ + C → R + C^−^˙

15 C˙^−^ → C_ox_˙

16 C_ox_˙ + R → R˙^+^ + P

R˙^+^ + R˙^−^ → R* + Ror

17 C_ox_˙ + R˙^−^ → R* + P

18 R* → A + *hv*

R represents the ECL emitter, C represents the co-reactant, C_ox_ is the reductive intermediate of the co-reactant, and P represents the product associated with C_ox_˙ reactions.

## 3. Fundamental apparatus

**Fig. 1:**
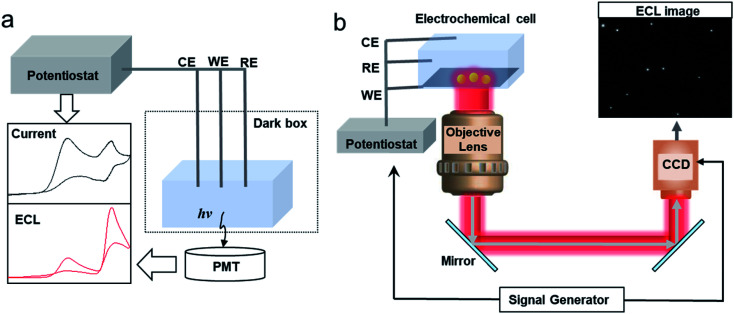
Schematic of (a) a setup of the conventional intensity based ECL detection system and (b) an ECL microscopy system for SEA measurement of nanoparticles (NPs).

For the intensity based ECL detection of single entities, the typical setup includes a photomultiplier tube (PMT), an electrochemical cell and an electrochemical workstation (Fig. 1a).^52^ Commercial instruments have been developed to synchronize the PMT and electrochemical workstation to record the light and electrical signals simultaneously. It should be noted that to pick up the signal of single entities, an ultra-micro electrode (UME) or a nanoelectrode with small capacitances, thin diffusion layers and fast response times has been applied in the study.^37^

The instrumental setup of ECL imaging (ECLM) is also simple. The basic setup of ECL microscopy includes an electrochemical cell, an electrochemical workstation and a bright field microscope equipped with a charge-coupled device (CCD) or an electron multiplying CCD (EMCCD) (Fig. 1b).^38,39,53^ A microscope objective with a high numerical aperture (N.A.) is preferred to increase the collection efficiency. A signal generator could be coupled to synchronize the CCD and the electrochemical workstation. After switching off the external light, a potential step or sweep voltammetry is applied to trigger the electrochemical reactions, while the CCD or EMCCD will capture the ECL image in real-time.

## 4. Single-molecule detection

**Fig. 2:**
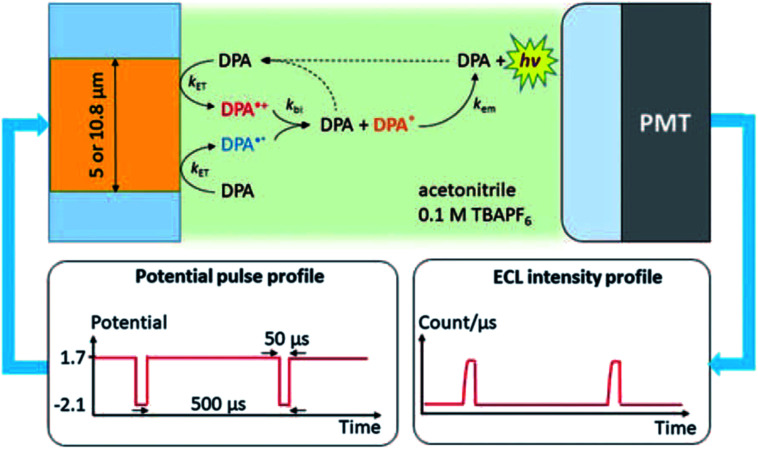
Schematic of the system used to measure ECL of individual DPA molecules. The Au UME is facing a PMT. A 20 femtoliters solution containing few tens of mM of DPA dissolved in acetonitrile with 0.1 M of tetrabutylammonium hexafluorophosphate (TBAPF6) is introduced into the gap under flow conditions. Potential pulses applied to the UME produce a cascade of reactions that lead to light emission measured by the PMT. Reproduced from ref. 54 with permission from the Royal Society of Chemistry, copyright [2020].

The first report of single-entity ECL comes from a single molecule. In 1995, Wightman's group reported the ECL reaction of 9,10-diphenylanthracene (DPA) in acetonitrile with potential pulses applied to an UME.^37^ The reaction follows the annihilation pathway. The gold UME is pulsed at *ca.* 1.8 kHz from 1.7 V to −2.1 V *vs.* Ag, potentials where molecules of DPA are reversibly converted to DPA^+^˙ and DPA^−^˙, which react with each other in the solution and produced excited states. A PMT is positioned at *ca.* 2 mm from the surface of the UME in order to collect the photons emitted in the reaction layer. These events were stochastic and followed the Poisson distribution. The experimental setup and the ECL mechanism are carefully elaborated by J. E. Dick and C. Renault (Fig. 2).^54^ When events during a single cathodic pulse are viewed with a greater temporal resolution (with 50 μs or shorter interval), photons generated from ECL are resolved. This pioneering work proves that ECL from individual molecules can be detected. The ability to observe individual ECL reactions opens new possibilities to determine the rate-limiting step of bimolecular reactions. From the single-entity measurements and relevant statistical analysis, the chemiluminescence quantum yield of the DPA was determined to be around 6%, in good agreement with ensemble measurements.

Recently, Jiang's group reported ECL detection of single-molecule microRNA.^55^ They synthesized a novel ECL probe, a cyclometalated dinuclear Ir(iii) complex, [Ir_2_(dfppy)_4_(imiphenH)]PF_6_ (imiphen = 2-(1*H*-Imidazole-2-yl)-1*H*-imidazo[4,5-*f*][1,10]phenanthroline; PF_6_^−^ = hexafluorophosphate), in which two {Ir(dfppy)_2_}^+^ units are bridged by an imiphenH^−^ ligand. The ECL emitter showed significantly enhanced ECL due to the synergistic effect, which could be mainly attributed to the dinuclear structure of the complex in which two Ir(iii) centers are bridged by an aromatic organic ligand. Theoretical calculation reveals that the enhanced ECL is associated with the two features of this complex, which are the S_0_–S_1_ excitation and the possible electronic coupling between two Ir(iii) centers. Based on the synergistic enhanced effect of ECL, this complex could be used to detect microRNA-21 at the single-molecule level, with detectable ECL emission from this complex intercalated in DNA/microRNA21 as low as 90 helix molecules. In other words, an ultra-sensitive ECL assay for single molecule detection requires not only advanced equipment, but also ECL probes with high luminescence efficiency.

## 5. Single particle detection

In recent years, ECL imaging of single micro/nano particles has flourished. Taking advantage of optical microscopy, individual nanoparticles, including noble metal,^30,56,57^ semiconductor^58^ and polymer nanomaterials,^59^ with diameters of tens to hundreds of nanometers could be traced by ECL simultaneously with high spatial resolution. Nanoparticle analysis, catalytic reactions and novel sensing platforms are all accessible by the ECL measurements. In addition, the micro/nano fabrication of microelectrodes and devices greatly promotes the ultra-sensitive sensing and mechanism study.

### 5.1 Imaging of static single particles

**Fig. 3:**
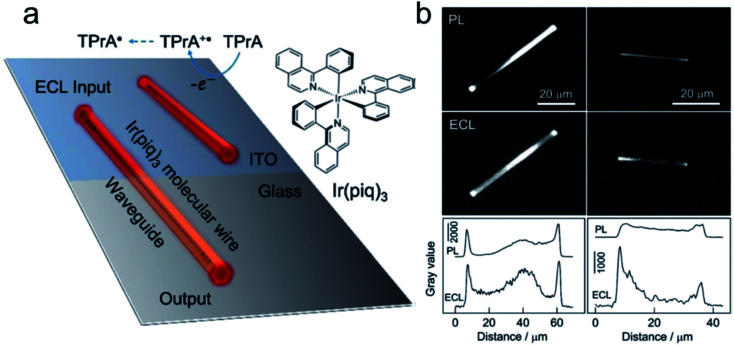
(a) Schematic illustration of the ECL waveguide (ECLW) in single molecular wires of Ir(piq)_3_ on a patterned ITO electrode; (b) PL and ECL images of single molecular wires of Ir(piq)_3_ on the ITO electrode in 0.1 M PB solution (pH 7.4) containing 0.1 M TPrA. ECL images were captured at +1.5 V. The exposure time was 60 s. The grayscale variation of PL and ECL along the longitudinal axis of two molecular wires shown in PL and ECL images. Reproduced from ref. 60 with permission from Wiley-VCH, copyright [2020].

The first report of ECL on single nanoparticles was provided by Bard's group.^59^ An ECL polymer, poly(9,9-dioctylfluoreneco-benzothiadiazole) (F8BT), has been synthesized into nanoparticles with an average diameter of 25 nm. Using TPrA as the coreactant, the ECL emission of F8BT nanoparticles has been triggered at a potential above *ca.* 1.8 V *vs.* Ag and observed under ECLM. *Via* the study of the relationship between the volume of the nanoparticle and ECL intensity, it shows that ECL from individual F8BT nanoparticles is produced within the volume of the nanoparticle instead of its surface. In addition, the observation of ECL trajectories reveals a deactivation mechanism that is dependent on the size of the nanoparticle. Most recently, Su's group reported the observation of an active waveguide of ECL in single crystalline molecular wires self-assembled from cyclometalated iridium(iii) complexes, namely tris(1-phenylisoquinoline-C^2^, N) (Ir(piq)_3_), which functions as both an ECL emitter and active waveguide.^60^ ECL generated under electrochemical control can be well confined to and propagate along the wire with a diameter of 1 μm to emit strongly at terminals (Fig. 3).

In addition to the observation of ECL nanoemitters, ECL offers a great opportunity to determine the electrocatalytic activity of noble metal nanocatalysts by monitoring the local electrocatalytic reaction rate toward ECL luminophores and coreactants. In 2015, Pan's group first imaged the local electrochemical activity of single gold nanoparticles with diameters from 30 to 300 nm by ECL, which was affected by the local chemical and charge transfer environment.^56^ In the ECL imaging, Ru(bpy)_3_^2+^ is used as the ECL luminophore and TPrA is used as the coreactant. The obtained ECL generation was unstable and vanished within several seconds because of the highly active AuNP surface that could be easily oxidized under positive potential. In the same year, Willets *et al.* reported ECL imaging of polymer coated micro-sized (length: 5–10 μm) gold nanowires.^57^ Using a mixture of polymers, including poly(3,4-ethylenedioxythiophene)-poly-(styrenesulfonate, PEDOT:PSS) and poly(vinyl alcohol), and PVA as the surface protectant, gold nanowires were quite stable during voltammetric cycles.^57^ They also discovered that the polymer thickness affected the sharpness of the ECL images.

**Fig. 4:**
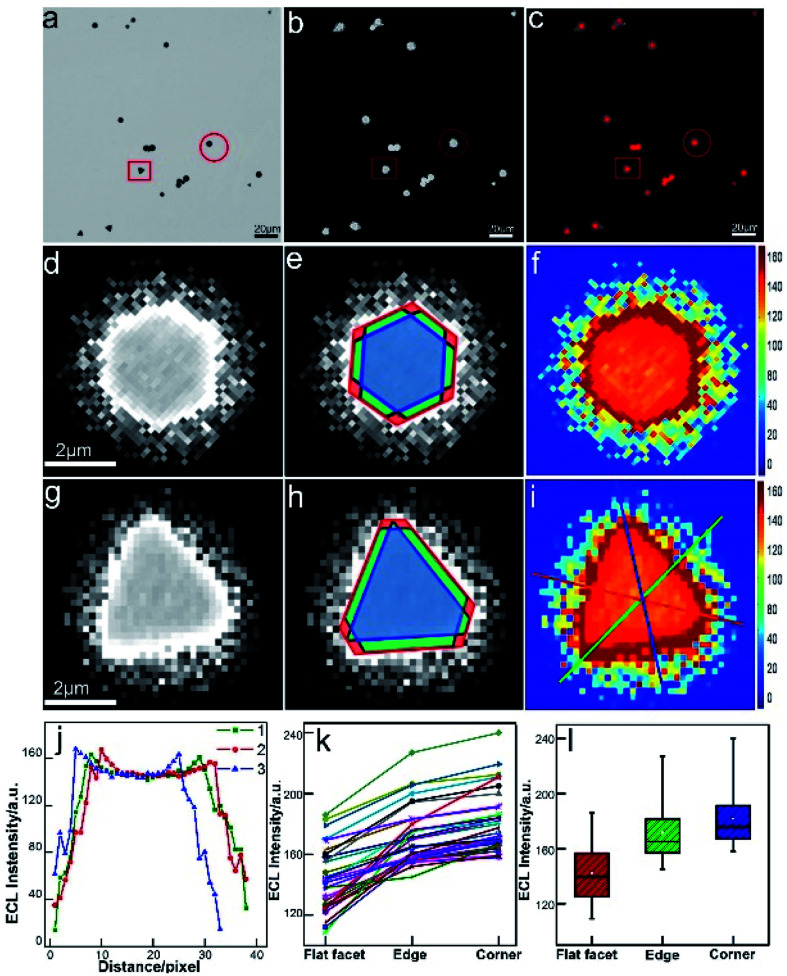
Spatial distribution of ECL intensity on single gold nanoplates. (a) Bright-field image, (b) ECL image (exposure time: 200 ms) and (c) false-color overlay of the bright-field and ECL images. (d and g) 3 magnified areas of the ECL image (b) marked by a red circle and square. (e and h) Divided regions of corners (red), edges (green), and the flat surface facet (blue) in the ECL images of (e and h). (f and i) 2D spatial distribution ECL intensity obtained by using Matlab. (j) ECL intensity gradients at three measuring lines marked in (i). (k) Statistical site specific ECL intensity of 35 nanoplates. (l) Box charts of the ECL intensities from corner, edge and flat facet regions. Reproduced from ref. 62 with permission from the Royal Society of Chemistry, copyright [2020].

The real-time and high-throughput ECLM could bring about more comprehensive understanding of electrocatalytic reactivity of bimetallic nanocatalysts. Our group employed ECLM to image the electrocatalysis of a novel bimetallic nanoparticle, Au–Pt Janus nanoparticles with a diameter of *ca.* 60 nm.^30^ Compared to the monometal, the Janus particle with the special heterogeneous structure exhibited enhanced ECL intensity and stability, indicating better catalytic efficiency. Based on the experimental results and digital simulation, it was concluded that concentration difference arose at the asymmetric bimetallic interface according to different electron-transfer rate constants at the opposite sides. The fluid slip around the Janus particle enhanced local redox reactions and protected the surface of the particle from passivation. The electrocatalytic activities of 1D Pd–Au nanorods (66 ± 4 nm in length and 20 ± 2 nm in diameter) with different bimetallic nanostructures were also studied at the single-particle level.^61^ As a result of the difference in heterogeneous electron transfer rate constants of Pd and Au, the dog bone-shaped Pd-tipped Au nanostructures showed obviously higher ECL intensity, while the two-faced Pd–Au Janus NRs exhibited better stability compared to monometallic Au NRs and Pd-covered Au NRs with a homogeneous surface. The results based on ECL analysis provide a reference for the synthesis of highly efficient bimetallic nanostructures with specific composition distribution. Furthermore, we extend our study to 2D nanomaterials for directly mapping out the heterogeneously distributed electrocatalytic activity on individual micro-sized (diameter of 1–4 μm) gold nanoplates.^62^ The *in situ* ECL imaging reflects the site-specific electrocatalytic activity within a single nanoplate (Fig. 4). Compared with the study using super-resolution fluorescence microscopy,^7^ ECLM allows for imaging with much higher temporal resolution (millisecond), which may lead to more comprehensive understanding of reactivity patterns of the single nanocatalyst.

In addition to the study of the constitution and structure of the nanocatalyst by conventional ECL reactions of Ru(bpy)_3_^2+^/TPrA and luminol (or its derivative)/H_2_O_2_ systems, ECLM can also provide a visualization approach to evaluate important electrocatalytic reactions concerning energy conversion.^58^ Zhu's group discovered that hydroperoxide intermediate OOH_(ad)_ generated on the surface of quantum dots (QDs) during the electrocatalytic water oxidation, which can react with luminol species as the coreactant and produce ECL emission.^58^ They compared QDs with different valence band positions (CdSe, CdTe, and CdSeTe) and found that CdSeTe displayed the highest ECL intensity due to more defects and a suitable valence band. Furthermore, the catalytic activities from different subparticle regions of ZnO crystal were studied by ECLM by the same group.^53^ Recently, they reported work on identifying hydrogen evolution reaction (HER) activities of single nanocatalysts based on ECLM.^63^ An ECL blinking phenomenon was observed at the single-nanoparticle level to directly monitor H_2_ nanobubbles generated from hollow carbon nitride nanospheres (HCNSs). Gas chromatography shows that the HER of HCNSs in the electrolyte containing 100 mM S_2_O_8_^2−^ produces 58.6 ppm H_2_ molecules. The generation, growth, and collapse of H_2_ nanobubbles could be identified by the ECL ON and OFF mechanisms. The power-law distributed durations of ECL ON and OFF states demonstrate multiple catalytic sites on a single HCNS. In addition, the ECL blinking phenomenon provides an explanation for the low cathodic ECL efficiency of semiconductor nanomaterials.

**Fig. 5:**
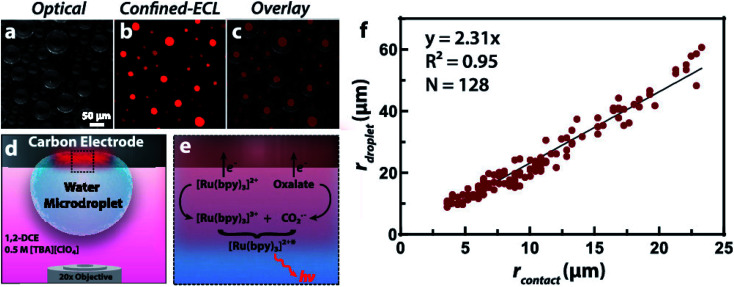
(a) Optical image of water microdroplets loaded with 10 mM Ru(bpy)_3_Cl_2_ (luminophore) and 50 mM sodium oxalate (coreactant) adsorbed to an inverted 1.5 mm radius glassy carbon electrode. (b) ECL signal generated by the application of 1.3 V *vs.* Ag/AgCl to oxidize both Ru(bpy)_3_^2+^ and oxalate, false-colored as red. The signal is confined to the water|electrode interface due to the insolubility of oxalate in the oil phase, producing a contrast at the water|1,2-dichloroethane interface and allowing a direct measurement of the contact radius. (c) Overlay of the optical and ECL images. (d) Schematic representation of the two-phase system where ECL is confined within the aqueous microdroplet. Tetrabutylammonium perchlorate ([TBA][ClO_4_]) acts as a supporting electrolyte and charge balance mediator by the transfer of ClO_4_^−^ into the droplet upon oxidation of the ECL reagents. (e) Schematic representation of the direct ECL reaction pathway. (f) Plot of the microdroplet radius as a function of the measured contact radius revealing a linear trend. A silver chloride wire and platinum wire served as the reference and counter electrode, respectively. The exposure time was 10 s. Reproduced from ref. 64 with permission from the American Chemical Society, copyright [2020].

To obtain more understanding of phase boundaries, Dick and co-workers described the measurements of imaging phase boundaries using ECL on glassy carbon electrodes.^64^ The selective imaging of the two-dimensional contact interface for individual micrometer water droplets suspended in 1,2-dichloroethane may be achieved by selectively dissolving the ECL luminophore and coreactant in the water microdroplets to make ECL reaction only proceed in the aqueous phase. These ECL imaging experiments capture the entirety of the water/electrode boundary to provide contrast with the oil phase, which allowed quantification of the boundary thickness of 9 ± 3 μm, the microdroplet contact radii, as well as the growth dynamics of electrogenerated O_2_ bubbles(Fig. 5). The fundamental knowledge of phase boundary chemistry based on ECL imaging of droplets will help the development in biology, nanoscience, synthesis, and energy storage and conversion.

### 5.2 Observation of single-particle impact

Single-entity measurements based on electrochemical methods have been widely reported to analyze nanoparticles, as well as to study the interfacial charge and electron transfer process. Bard's group reported the collision between UMEs and toluene droplets suspended in water.^65–68^ The amperometric current and ECL intensity were recorded simultaneously. The droplets are synthesized by adding toluene to water with a stabilizing surfactant. Their size typically ranges between 300 and 1800 nm in diameter. These droplets can be loaded with a redox active material that is soluble in one phase but not the other. Bard and co-workers loaded toluene droplets with a rubrene and TPrA to produce ECL upon oxidation of the two reagents when the droplets collide with an electrode polarized at 1.1 V *vs.* Ag/AgCl. The current and ECL signals were recorded simultaneously, indicating that individual collision events could be monitored by both electrochemical and optical methods.^68^ They also demonstrated the possibility of observing single metal (Pt) NP (∼4 nm diameter) collision events with a UME using an ECL pair of Ru(bpy)_3_^2+^/TPrA.^69^ An individual collision event produces a unique photon spike with amplitude and frequency that can be correlated with the size and concentration of the Pt NPs. The catalytic ECL amplification allows the study of the dynamic process and kinetics of the heterogeneous electron transfer at the single particle level.

**Fig. 6:**
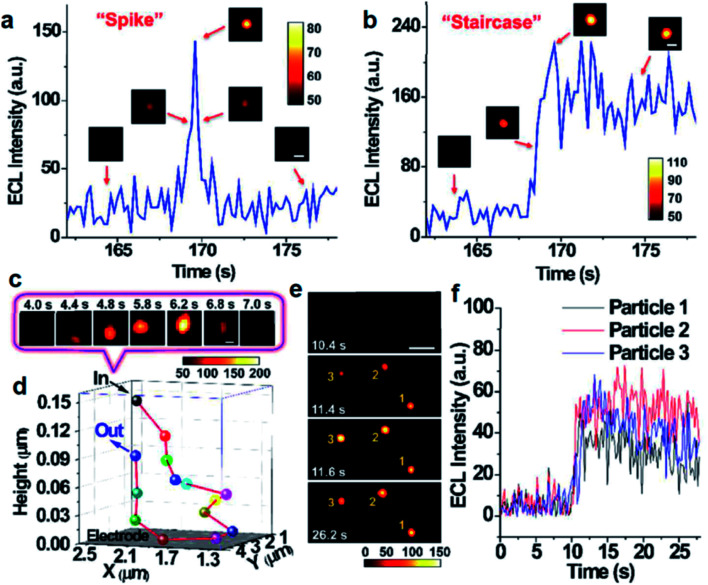
Single ECL spike (a) and staircase (b) signals during the collisions processes. (insets) ECL snapshots of individual nanoparticles during a typical collision process. Constant potential: 1.4 V. Scale bars (white), 2 μm. Exposure time: 0.2 s. (c) ECL snapshots of individual nanoparticles during an elastic collision with Brownian motion. Scale bars (white), 1 μm. Exposure time: 0.2 s. (d) 3D collision trajectory of single nanoparticle in (c). (e) ECL snapshots of three RuDSNs colliding with the electrode simultaneously. Scale bars (white), 5 μm. Exposure time: 0.2 s. (f) ECL intensity *vs.* time curves of three RuDSNs in (e). Reproduced from ref. 70 with permission from the Royal Society of Chemistry, copyright [2018].

In addition to the intensity based single-particle impact observation, ECLM is able to investigate some dynamic collision behaviors of single nanoparticles owing to the spatiotemporal resolution. Zhu's group used ECLM to dynamically image collision electrochemistry of single Ru(bpy)_3_^2+^-doped silica nanoparticles (RuDSNs) with average diameters of 90 nm, 420 nm and 1.5 μm (Fig. 6).^70^ The ECL trajectories of single RuDSNs as a function of electrode potential demonstrated the two ECL mechanisms of RuDSNs at the single-particle level. They synthesized RuDSNs with three different sizes to demonstrate the relationship between the ECL efficiency and particle sizes. The spatial resolution of ECLM enabled the monitoring of simultaneous collisions of multiple nanoparticles, which cannot be studied using the ECL intensity-based technique.

### 5.3 Micro/nano technology based sensing platform

Micro/nano fabricated electrochemical devices offer a highly controlled geometry, which confine the volume of reactions to the micro/nanoscale and enable a greatly amplified electrochemical signal.^71^ Microfabricated bipolar electrode (BPE), nanofluidic device and microbeads based ECL systems could be utilized as efficient sensing platforms toward ultra-sensitive detection and mechanism study.

**Fig. 7:**
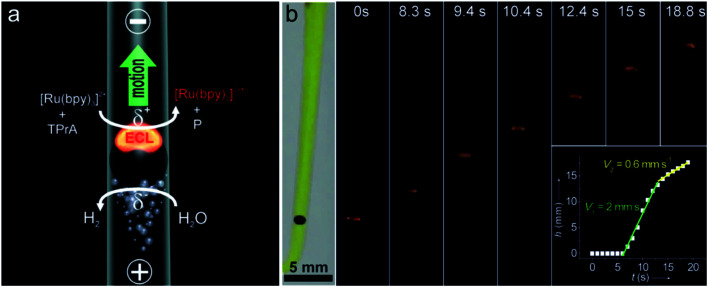
(a) Asymmetric light-emitting electrochemical swimmer. Simultaneous reduction of H_2_O at the cathodic pole (bottom of the bead) and oxidation of ECL reagents at the anodic pole (top of the bead) induce both motion and light emission from the bead in a glass capillary. P corresponds to a side product of the TPrA radicals formed during the ECL process. (b) Levitation of a light-emitting GC bead. Series of optical images showing a GC bead emitting ECL at different times during its motion. The bead was placed in a U-shaped cell, filled with 100 mm PBS buffer containing 0.5 mm Ru(bpy)_3_^2+^, 100 mm TPrA, and a few drops of the surfactant. It was exposed to an external electric field of 25.5 V cm^−1^. The left image was taken under white light and the other images were taken in the dark. Inset: plot showing the change in height *h* of the bead as a function of time *t*. Reproduced from ref. 76 with permission from Wiley-VCH, copyright [2012].

A BPE is an electrically conductive material that promotes electrochemical reactions at its poles.^72^ When sufficient voltage is applied to an electrolyte solution in which a BPE is immersed, the interfacial potential difference between the BPE and the solution drives the redox reactions. The wireless feature of BPEs makes large arrays of electrodes able to be controlled by a single power supply. In 2008, Crookes' team reported a microfabricated BPE array consisting of 1000 gold electrodes with 500 μm length and 50 μm width, using ECL as the optical readout.^73^ When a sufficient driving voltage is applied, ECL is produced at the anodic pole of each BPE. Massively parallel arrays of BPEs can be used for a variety of sensing and screening applications.^74^ In addition, bipolar electrochemistry enables mobile electrodes and microswimmers, which are able to move freely in solution.^75^ Sojic and Kuhn showed that BPEs could operate as a wireless electrochemical swimmer.^76^ By coupling ECL emission and H_2_ bubble production, a single light-emitting bipolar electrochemical swimmer was demonstrated. As depicted in Fig. 7a, a glassy carbon bead was putting in a capillary. The asymmetric electroactivity induced by bipolar electrochemistry generates simultaneous motion and light emission of the swimmer. The polarization voltage induced on the bead is proportional to the external electric field and the diameter of the bead. The speed of the swimmer was influenced by the formation and escape of the bubbles (Fig. 7b). Along the same line, they reported the local sensing and reporting of glucose in a concentration gradient explored by the ECL swimmer.^77^

Furthermore, the same group demonstrated 3D ECL using a BPE to electrochemically address millions of micro- or nano-objects simultaneously in a wireless way.^78^ Each single object (microbead or multi-walled carbon nanotube) acts as an individual emitter of an electrochemically generated luminescence signal, and their collective behavior enables strong light emission in the whole volume of the solution. Such a bulk emission of light opens the door for new applications of ECL such as high-sensitivity analysis or optical tracking of nanomotors. Using such a micro-fabricated device, they reported the dual biosensing concept of simultaneous quantitative determination of both glucose and choline over a wide concentration range.^79^ Lately, they presented a general strategy that combines both external magnetic and electric fields with ECL emission in a BPE device.^80^ A gold-coated iron wire (150 μm diameter and 20 mm length) was applied as the BPE. External magnetic and electric fields induce the rotational motion of the wire and its bipolar polarization and local generation of ECL emission, respectively. During the rotation, the motion is tracked by ECL intensity variations as a function of the orientation of the conducting wire in the electric field. Such an approach opens exciting prospects for integrating new functions such as imaging and sensing capabilities, as well as dynamic enzymatic detection or immunoassays.

Microfabricated nanodevices, combining the merits of precise control of the geometry and strong confinement of analytes, offer an opportunity for the highly sensitive sensing and mechanistic study.^71,81^ Sojic, Mathwig and co-workers reported a transparent nanofluidic electrochemical device with two individually addressable electrodes separated by a distance of 100 nm.^81^ The nanoscale distance between electrodes results in a short diffusion timescale between them and gives rise to highly amplified currents through electrochemical redox cycling. On the basis of such a device, enhanced annihilation ECL light emission of attomole luminophore quantities is recorded. Lately, Su's group reported the measurement of an ECL layer to decipher reaction mechanisms by imaging microtube electrodes using ECLM.^82^ They fabricated microtubes of three sizes with outer/inner diameters as *ca.* 2.3/1.4 μm, 5.4/4.5 μm, and 10.4/9.0 μm, respectively. For the classical Ru(bpy)_3_^2+^/TPrA system, the ECL layer was resolved to vary from about 3.1 μm to >4.5 μm upon increasing the concentration of Ru(bpy)_3_^2+^, while for the Ru(bpy)_3_^2+^/2-(dibutylamino)ethanol (DBAE) system, the ECL pattern remains unchanged with a constant TEL of about 2.1 μm. The results prove that the diffusion distance of DBAE-derived radicals is shorter that of TPrA radicals in solutions. The detection of the ECL layer based on microelectrodes is capable of deciphering the ECL mechanisms and provides insights into ECL reactions.

**Fig. 8:**
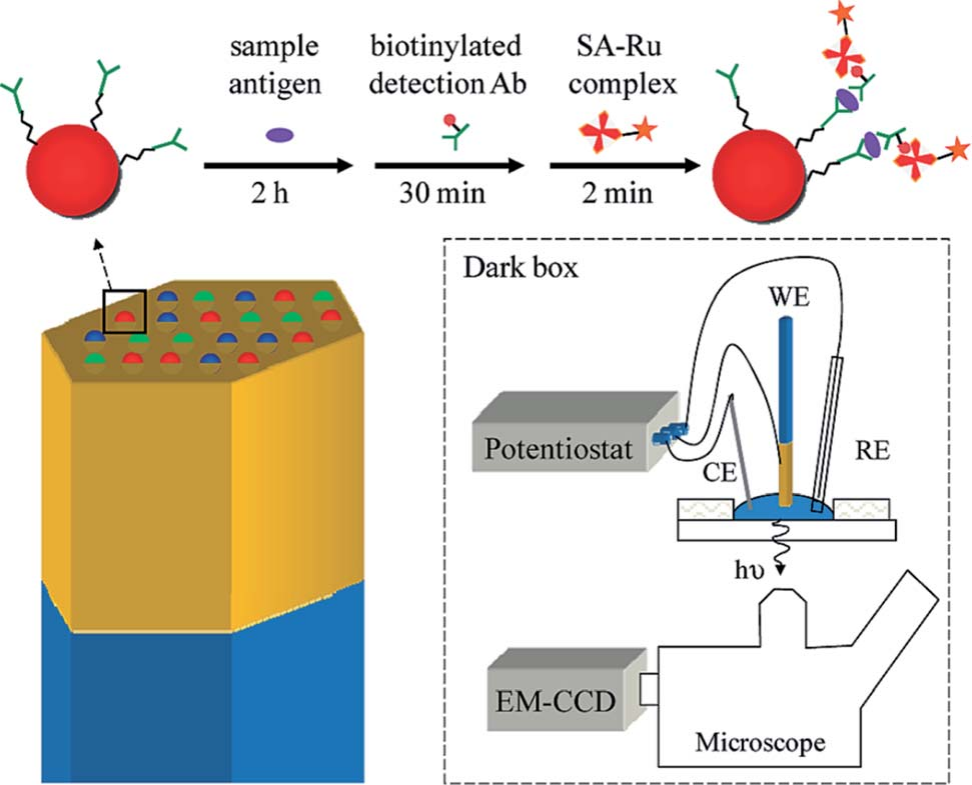
(Top) A sandwich immunoassay is performed by exposing antibody-functionalized microbeads to three solutions: (1) an antigen containing sample, (2) biotinylated detection antibodies, and (3) streptavidin modified with a Ru(bpy)_3_^2+^ complex (streptavidin(SA)-Ru). The beads are housed in microwells created from an etched gold-coated fiber-optic bundle. The gold coated fiber bundle acts as the working electrode (WE) for ECL. CE and RE refer to the counter electrode and reference electrode. Reproduced from ref. 83 with permission from the American Chemical Society, copyright [2009].

As is commonly known, microbeads are ideal labels in ECL-based sensors. In 2009, Sojic's group reported multiplexed sandwich immunoassays at the single bead level using ECL imaging.^83^ The multiplexed ECL platform consists of 3.1 μm Ru(bpy)_3_^2+^-modified polystyrene (PS) microspheres loaded into the wells of an electrode, prepared from etched fiber optic bundles coated with gold (Fig. 8). The surfaces of the microspheres were modified with three antibodies. A CCD camera can simultaneously image the PS microspheres modified with different antibodies for recognition with the corresponding targeted antigens. With the aid of fluorescence imaging, three types of PS microspheres were differentiated according to the inherent different fluorescence intensities. This approach should enable the analysis of dozens of analytes simultaneously.

**Fig. 9:**
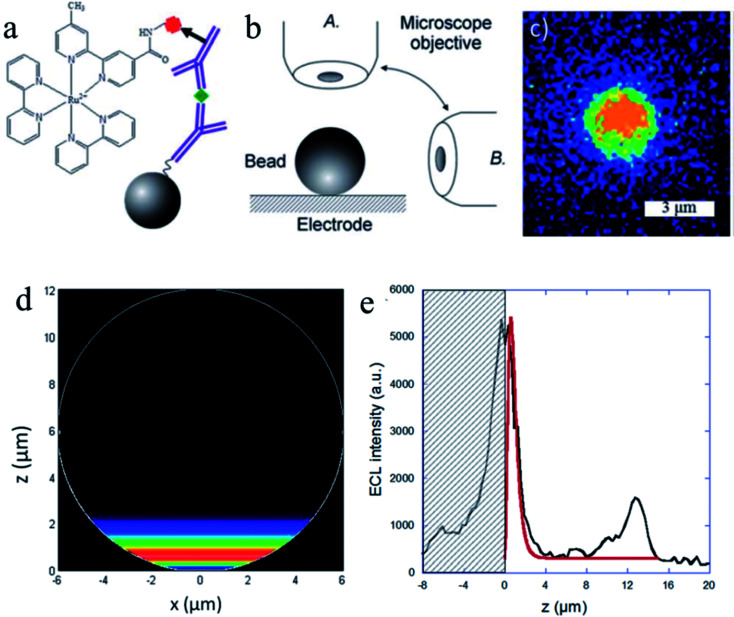
(a) Sandwich immunoassay with PS beads. (b) Schematic representation of both of the optical configurations used to image the functionalized bead: top-view (A) and side-view (B). (c) ECL imaging of a single 3 μm bead using the top-view configuration. ECL images were acquired over a 6 s exposure-time at a potential of 1.1 V *vs.* Ag/AgCl/KCl in a PBS solution containing 100 mM TPrA (pH = 7.4). (d) Side-view of the simulated distribution of the generated Ru(bpy)_3_^2+^* excited state (*i.e.* ECL intensity) on the surface of a 12 μm bead. (e) Comparison of the experimental (black line) and simulated (red line) ECL intensity profiles at the level of a single bead. The experimental data correspond to the PS bead in Fig. 3a recorded at 1.1 V. The ECL signals are simulated with a value of 2920 s^−1^ for the TPrAc^+^ deprotonation rate constant. The hatched zone represents the reflection of the ECL light on the electrode surface. Reproduced from ref. 87 with permission from the Royal Society of Chemistry, copyright [2014].

Understanding the ECL mechanisms is important toward the design of efficient sensors with optimum generation and collection of light.^84–86^ To explore the ECL mechanism of Ru(bpy)_3_^2+^ with two efficient coreactants (TPrA and DBAE), Sojic and co-workers reported ECL measurements (Fig. 9) on individual polystyrene microbeads of 12 μm diameter.^87^ Ru(bpy)_3_^2+^ was attached to the surface of the beads *via* sandwich immunoassay or a peptide bond. They used an orthogonal side-view configuration to directly image the ECL distribution longitudinally. Mapping the ECL reactivity on the microbead demonstrates the generation of the excited state at a micrometric distance (3 μm) from the electrode by reaction of surface-confined Ru(bpy)_3_^2+^ with diffusing TPrA radicals. The TPrA˙^+^ lifetime was determined from the ECL profile. On the other hand, DBAE generates very low ECL intensity in the bead-based format, suggesting that DBAE-derived radicals had much less lifetime and cannot propagate such a long distance as TPrA-derived radicals. The 3D imaging approach based on single microbead provides insights into the ECL mechanistic route operating in bioassays and on the optical effects that focus the ECL emission. Lately, spatially resolved ECL was further reported by the same group using microbeads.^88^ To control the thickness of the ECL-emitting region, they exploited the buffer capacity of the solution to tune the rate of the reactions involved in the ECL generation. By mapping the luminescence reactivity at the level of a single bead, precise control of the ECL light distribution was obtained, which provided insights into the ECL mechanism.

## 6. Single-cell detection

Because of the broad cellular heterogeneities, single-cell detections are highly desirable, since they provide accurate information about cellular processes avoiding ensemble average and show promising potential for gaining insight into fundamental biological processes and early diagnosis in clinic medicine.^89–91^ Compared to the main optical detection strategies such as fluorescent labeling, ECL detection of single living cells proved to be a new method to study the surface chemical composition and local activities of cells with a high signal to noise ratio. Although the analysis of a single cell by intensity and imaging based ECL is a very recent approach, a lot of meaningful studies have been reported.

### 6.1 Intensity based single cell analysis

The intensity-based ECL using a PMT instead of microscopy was used to capture the optical signal. It requires an extremely high sensitivity to reach the detection of single cell. Yang's group reported single-cell analysis using solid-state zinc-coadsorbed carbon quantum dots (ZnCQDs) as an ECL probe to improve the sensitivity of the assay.^92^ A remarkable 120-fold enhancement of the ECL intensity was obtained. Hyaluronic acid -functionalized ZnCQD probes were used to label a single breast cancer cell to recognize CD44 on the cell surface. This strategy exhibited a good analytical performance toward the analysis of MDA-MB-231 and MCF-7 single cells with a linear range from 1 to 18 and from 1 to 12 cells, respectively. Furthermore, they studied the adhesion of a single platelet to endothelial cells using an Au@DL-ZnCQD (DL = double layer) nanocomposite as the ECL signal reporters to label the single damaged endothelial cell.^93^ The ECL signal is sensitive to the adhesion of a single platelet adhered to the cell. The adhesions of activated and unactivated platelets were compared at the single cell level. Recently, this group reported a single-cell sensor with a spatial architecture.^94^ An 11-mercaptoundecanoic acid (MUA)-spaced sensing interface was designed to prop up the cell, leaving a space under the cell for nanoprobe labeling. The ECL intensity increased with increasing carbon chain length of MAs (MA-C*n*, *n* = 2, 4, 6, and 11) and reached the maximum when *n* = 11. With MUA modified, the sensor was able to enhance an ECL intensity of 37.5 ± 3.9%, indicating the enhanced accuracy for single-cell analysis. In 2016, our group reported an open bipolar electrode (BPE) sensing platform-based temporal detection of a single cell.^95^ The cathode of the BPE acted as the sensing pole, which was modified with anti-CEA IgG to capture MCF-7 cells. The anode was modified with Au@Ag coreshell nanoparticles, and ECL emitters were added to the corresponding reservoir. After the external potential was applied on the BPE, the Ag shell on the Au core started to dissolve and the ECL of luminol started to recover. The ECL recovery time was related to the number of cells captured on the cathode. This temporal detection method achieved a 10 times lower detection limit compared with conventional intensity-based ECL-BPE methods.^95^ The detection limit could be down to a single-cell level.

**Fig. 10:**
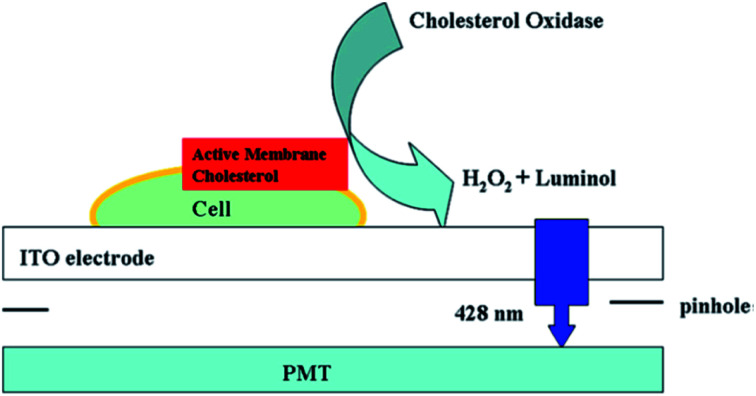
Setup of luminol electrochemiluminescence for the analysis of active cholesterol in the plasma membrane in single cell analysis. Reproduced from ref. 52 with permission from the American Chemical Society, copyright [2013].

To directly capture the ECL signal from one cell, Jiang's group redesigned the signal acquisition system by placing a pinhole (the diameter is 100 μm) below the electrode; therefore, only one cell was exposed to the PMT (Fig. 10).^52^ It was utilized to analyze active cholesterol in the plasma membrane in single mammalian cells. The active membrane cholesterol was reacted with cholesterol oxidase to generate hydrogen peroxide on the electrode surface, which induced a measurable ECL. Twelve single cells were analyzed individually, and a large deviation in the luminance ratio was observed, indicating the cell heterogeneity on the active membrane cholesterol. Using a similar strategy, other biomolecules on single cell membranes could also be determined.^96–99^ Later, they improved the pinhole-based system and prepared a high throughput multi-microelectrode array, which consisted of eight microwells.^100^ Each of the microwells could only contain one cell. Controlled by a multiplexer, the voltage was applied to the eight microelectrodes sequentially, while the luminescence of each microelectrode was recorded with a PMT accordingly.

### 6.2 Imaging-based single-cell analysis

ECLM with high spatial and temporal resolution can be utilized to monitor cellular physiological processes and protein expression at the single-cell level. Research studies focused on both the visualization and quantification of intracellular molecules and the visualization of cellular surface structures and proteins.

In 2015, Jiang's group reported luminol ECL imaging of living cells.^101^ An upright optical configuration coupled with double potential mode was applied. HeLa cells were incubated on the ITO electrode. The adherence of cells on the ITO electrode slows down the diffusion of the luminol analog; therefore, the cell appears dark while the electrode surface without cells is bright, leaving a negative image of cells. The simultaneous recording of luminescence from multiple cells offered information about the efflux of hydrogen peroxide and the amount of active membrane cholesterol. To further improve the throughput of single cell analysis, they fabricated a cell-sized photoresist-based microwell (30 μm in diameter and height) array containing 64 microwells for simultaneous detection of multiple cells.^102^ The parallel measurements of intracellular glucose and cholesterol were conducted by the introduction of Triton X-100 into the buffer with oxidase, which broke the plasma membrane and released the cytosol in the microwells for the ECL imaging. On the basis of the locally generated H_2_O_2_ or reactive oxygen species (ROSs), other biomolecules and cellular physiological processes have been monitored at the single-cell level. Zhang's group reported ECL imaging of microRNA-21 in a single cancer cell based on the release of ROSs as the coreactant of luminol.^103^ Zhu's group developed a potential-resolved ECL method for apoptosis diagnosis.^104^ The decreasing expression of the epidermal growth factor receptor (EGFR) and increasing phosphatidylserine (PS) eversion on the cell membrane were sensitively detected by the ECL probes.

**Fig. 11:**
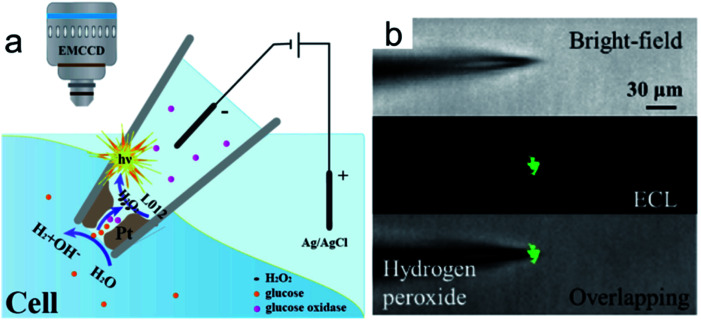
(a) Representation of the bipolar ECL detection of the porous Pt deposit inside the nanopipette, which is inserted into the cytosol for intracellular wireless electroanalysis. (b) Bright field, ECL, and overlapping images from the pipette tip to detect hydrogen peroxide outside the nanopipette; the concentration of hydrogen peroxide is 10 mm. Reproduced from ref. 105 with permission from Wiley-VCH, copyright [2020].

To improve the spatial resolution of ECL imaging, efforts have been devoted on the limitation of the diffusions of luminophores and the coreactants. Microelectrodes and nanochannels have been applied to confine the species generated in ECL reactions.^71,81,82^ Recently, Jiang and co-workers reported a nanopipette for intracellular analysis of single cells (Fig. 11a).^105^ They constructed an open bipolar system using porous Pt to deposit at the tip of a nanopipette (300 nm in diameter). The voltage drop is confined within the nanopipette tip. The presence of the electric field permits the sorting of various molecules from the intracellular cytosol into the nanopipette. Under this electric field, the intracellular molecules could react with the luminol analogue and produce ECL emission inside the nanopipette (Fig. 11b). The intracellular concentrations of hydrogen peroxide, glucose, and sphingomyelinase have been measured *in vivo*.

**Fig. 12:**
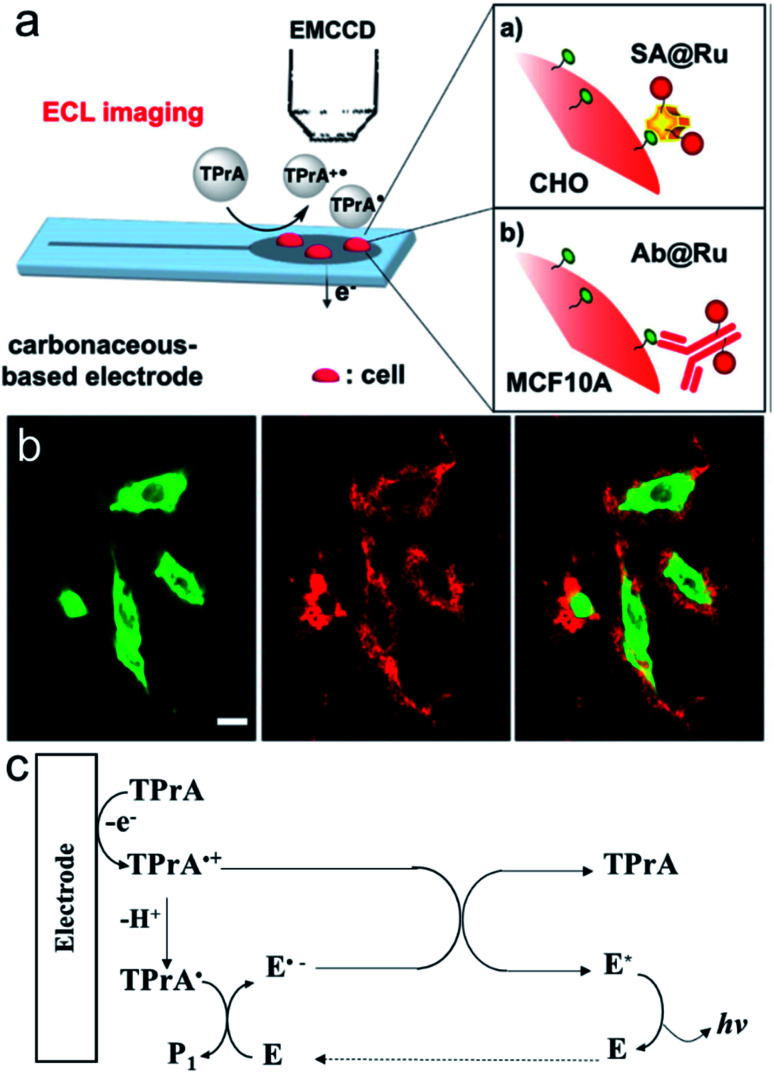
(a) Schematic principle for the ECL imaging of single cells. (b) PL (green), ECL (red) and overlay of the PL (green) and ECL (red) images of CHO cells grown on a GC electrode. SA(streptavidin)@Ru labels were attached to the biotinylated proteins of the cellular membrane. ECL was recorded in phosphate buffer solution (pH = 7.4) containing 100 mM TPrA by applying 1.35 V. Scale bar: 20 μm. (c) ECL mechanism involved in this work (E represents Ru(bpy)_3_^2+^). Reproduced from ref. 106 with permission from the American Chemical Society, copyright [2017].

Surface-confined ECL microscopy was first reported by Sojic's group to image single cells and their membrane proteins.^106^ The ECL fluorophore Ru(bpy)_3_^2+^ was linked to the plasma membrane or a specific antigen on the membrane by immune recognition to localize the ECL generation in close proximity to the electrode surface (Fig. 12a). As shown in Fig. 12b, the ECL signal is different from the photoluminescence signal. Compared with photoluminescence, ECL is observed only on the borders of the cells. The difference can be explained by the mechanism of ECL generation, in which only TPrA is oxidized on the electrode (Fig. 12c). The intermediates, TPrA˙ and TPrA˙^+^ exist in the vicinity of the electrode and their diffusion distances are limited because of their short lifetime. The dark area observed at the center of the cells on the ECL image indicates that the products of TPrA oxidation cannot access this central area. Later, they used Triton X-100 to treat the cells to increase the permeabilization of the membrane. Well-defined ECL imaging of the cell without any dark area was obtained.^107^

ECL imaging of antigens or proteins on the cell membrane usually requires ECL emitters as the labels. Alternatively, label-free techniques are more attractive due to the more friendly operation procedures. Zhang *et al.* reported ECL-based capacitance microscopy for label-free assays at the single-cell level for the first time^.^^108^ In their work, a square wave voltage with high frequency was applied to the working electrode. The authors claimed that both the local capacitance and the frequency of applied pulse voltage affected the local potential drop across the double layer (*V*_dl_). Upon the binding of the nonconductive antibody, the local capacitance decreases, resulting in a higher potential drop across *V*_dl_ in these regions. Under conventional low-frequency pulse voltage, the slight change in *V*_dl_ can hardly be detected. On the other hand, using a high-frequency (1.5 kHz) voltage, a highly sensitive detection of carcinoembryonic antigen (CEA) was achieved, with a detection limit as low as 17 ag. Su's group also reported an interesting study about the label-free imaging of cell–matrix adhesions and collective migration of single cells based on the surface-confined ECL microscopy.^109^ Cell–matrix adhesions form close contacts with the electrode surface, thus locally blocking both the ECL reaction and the diffusion of luminophores, which appear as a dark area under ECLM. The dynamic variations of the cell–matrix adhesions of living cells were recorded in the label-free manner.

**Fig. 13:**
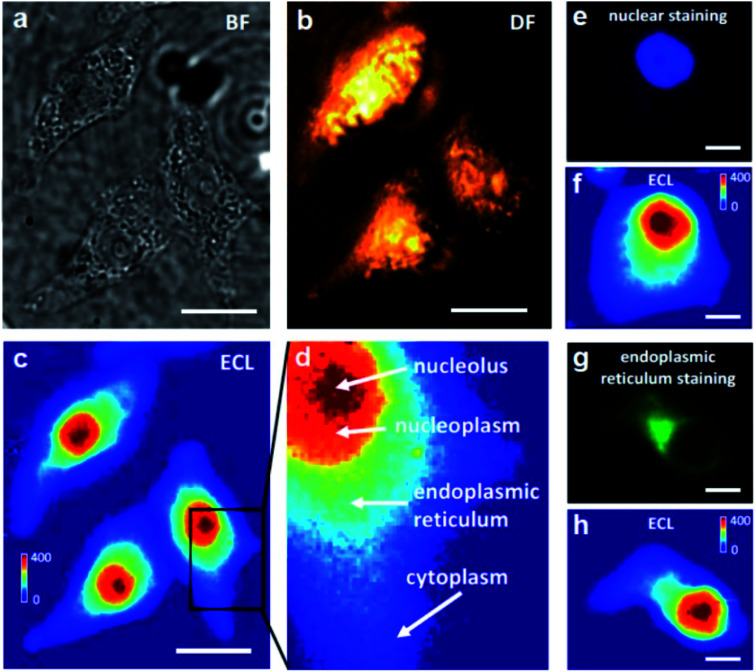
Bright-field (BF) (a), dark-field (DF) (b) and ECL (c) images of three adherent and fixed HeLa cells on an ITO electrode. The BF and DF images were captured using transmission and oblique incidence light sources, respectively. When a constant 1.3 V (*vs.* Ag/AgCl) was applied to the ITO electrode in 10 mM PBS (pH 7.4) containing 400 μM Ru(bpy)_3_^2+^, frame-by-frame ECL sequence images (exposure time: 1 s) were recorded on the EMCCD and an integrated ECL image of 400 original ECL serial images is shown in (c). Scale bar (white) is 20 μm. (d) A zoom-in ECL image of a single cell marked with the black box in (c). The red, orange, cyan and blue regions represent the nucleolus, nucleoplasm, endoplasmic reticulum and cytoplasm, respectively. (e, f) Fluorescence image of nuclear staining with DAPI and the corresponding ECL image. (g, h) Fluorescence image of endoplasmic reticulum staining and the corresponding ECL image. Scale bar (white) is 5 μm. Reproduced from ref. 111 with permission from Wiley-VCH, copyright [2021].

A coreactant-embedded ECL strategy was reported by Ju's group to image the membrane protein of a single living cell.^110^ Diethylamine conjugated polymer dots (TEA-Pdots) have been designed and synthesized, which could produce ECL emission without the need for a coreactant in the solution. Specific protein expression on the cell surface could be determined using this strategy. Later, adopting a similar concept, Ma *et al.* reported a bio-coreactant-enhanced strategy, which realized direct ECL imaging of the intracellular structure and dynamic transport.^111^ In their work, intracellular biomolecules with reductive amine moieties have been utilized as the coreactants to drive the cascade ECL reactions with Ru(bpy)_3_^2+^, which was used as the electrochemical molecular antenna to connect extracellular and intracellular environments. The dynamic transport of the ECL probes in the different cellular compartments unveils the heterogeneous intracellular diffusivity correlating with the actin cytoskeleton. The cells display a hierarchical ECL spatial distribution, which is highly relevant to the local concentration of intracellular DNA (chromosomes) that provides highly efficient coreactants to enhance ECL emission (Fig. 13). In addition to mapping the intracellular structures and measuring relevant events, the autophagy involving DNA oxidative damage is determined by nuclear ECL signals without using labels. They also applied bio-coreactant enhanced ECL microscopy to image bacterial colonies and liver tissue, indicating the practicability of this method in the study of relevant biological events.

Most recently, Dan, Sojic and co-workers reported the effect of photobleaching on ECL of single cells for the first time.^112^ Streptavidin-modified Ru(bpy)_3_^2+^ was used to label the plasma membrane of Chinese hamster ovary (CHO) cells. Specific regions of the cells were excited and photobleached sequentially with different laser powers, and then imaged by PL and ECL. ECLM demonstrates a linear correlation between the ECL decrease and the PL loss because of the photobleaching of Ru(bpy)_3_^2+^ immobilized on the cell membrane. On one hand, the influence of photobleaching effects in ECL may guide the design of efficient fluorophores for ECL imaging. On the other hand, combining ECL with photobleaching may provide a new approach to study the molecular dynamics and mobility within single cells.

## 7. Conclusions and outlook

Single-entity analysis, as an emerging area, has become the forefront of analytical chemistry. Starting from the first report of single-molecule detection in 1995 using ECL as the measuring technique,^37^ ECL has been rapidly adopted in the detection of individual things and separate individual response from the bulk. In this review, we conclude the history and recent development that has been made in single entity detection. Taking advantage of micro/nano fabrication technology and microscopy, the determination of the structure of nanomaterials, the study of catalytic reactions, resolving of ECL mechanisms, and the construction of novel sensing platforms are all accessible. Meanwhile, ECL nanopipettes, surface confined ECLM, and label-free and coreactant-embedded ECL strategies have been developed, which provided sensing platforms for measuring the cellular physiological processes, as well as the protein expression at a single-cell or even subcellular level. Clearly, the great improvement of ECL instruments and analytical methods provide opportunities for single-entity analysis.

For intensity-based ECL, combining a photomultiplier tube and single-photon counter, an extremely weak signal from single-entity could be obtained. For the new comers to this field, microfabrication of micro/nano electrodes with reproducible morphology and nanofluidic devices with a highly controlled nanochannel geometry might be the challenge. For real-time analysis of single-entities with high spatial and temporal resolution, ECLM is the better choice for researchers. Although ECL is a powerful tool with near zero background, during the imaging, the background from the substrate electrode and the diffusion layer of ECL could influence the observation, which could be of concern to beginners.

A significant present challenge for the detection of single entities using ECL is to push the limits of ECL instrumentation, which is also the most exciting and hardest part. Until recently, entities with diameters of about tens of nanometers could be observed under ECLM. However, the spatial resolution (∼200 nm) and temporal resolution (∼μs) are not enough for imaging the fine structure within a nanoparticle, as well as monitoring transient processes during a chemical reaction or a cell activity. Instrumentation advances are always pivotal to SEA.

In addition, the development of novel ECL reagent systems may greatly improve the intensity and the imaging resolution. In recent years, nanocrystals and clusters,^113,114^ aggregation-induced ECL polymers^115–118^ or near-infrared materials,^119,120^ with high ECL efficiency, stability, and biocompatibility have been reported, which are expected to replace the conventionally used Ru(bpy)_3_^2+^/TPrA pair and offer the opportunity to improve SEA study.

Understanding the relevance between many single-entity measurements and the impact of a single entity on the ensemble are very important, which make this area meaningful. Combining the inherent advantages of electrochemistry and optical detection techniques, ECL detections provide both highly sensitive and high-throughput single-entity measurements that could provide more information. Undoubtedly, the research in this field will continue to grow exponentially. Beyond technological progress and method development, single-entity study based on ECL will help people learn more about science and nature.

## Conflicts of interest

There are no conflicts to declare.
